# Nationwide Study on the Cervical Cancer Screening Pathway in Estonia

**DOI:** 10.1002/ijc.70466

**Published:** 2026-04-09

**Authors:** Aleksandra Šavrova, Helen Jakoby, Anna Tisler, Kaire Innos, Ülo Maiväli, Jana Jaal, Anneli Uusküla

**Affiliations:** ^1^ North Estonia Medical Centre Women's Clinic Tallinn Estonia; ^2^ Institute of Family Medicine and Public Health University of Tartu Tartu Estonia; ^3^ National Institute for Health Development Tallinn Estonia; ^4^ Institute of Clinical Medicine University of Tartu Tartu Estonia

**Keywords:** cervical cancer screening, human papillomavirus, loss to follow‐up, screening uptake

## Abstract

Effective cervical cancer screening and progress toward WHO elimination targets depend on complete follow‐up for women with positive primary screening tests. However, real‐world data on this critical step in hrHPV‐based screening programs in Eastern Europe are scarce. We conducted a nationwide, population‐based retrospective cohort study in Estonia, analyzing records from the Estonian Health Insurance Fund and Population Register. We included 44,282 women aged 30–65 who were invited to hrHPV‐based screening between January 1, 2021, and December 31, 2022, with follow‐up through March 31, 2024. Screening attendance was 45.7% (*n* = 20,242; 95% CI: 45.2%–46.2%), with 8.0% (*n* = 1615; 95% CI: 7.6%–8.4%) testing hrHPV‐positive. A substantial loss to follow‐up was observed: 57.7% of hrHPV‐positive women did not undergo repeat hrHPV testing, colposcopy, or any post‐colposcopy care within 12 months. Among those referred for further diagnosis, colposcopy was performed in 77.9% within 6 months. Treatment for High‐Grade Squamous Intraepithelial Lesion (HSIL) cases was high (85.5%; *n* = 124; 95% CI: 78.7%–90.5%), mostly within 3 months. Predictors of lower follow‐up adherence included older age and residence in South Estonia. Suboptimal screening uptake and high loss to follow‐up among hrHPV‐positive women are significant barriers to effective cervical cancer prevention in Estonia. These challenges mirror those seen in many organized screening programs globally. Prioritizing strategies to enhance follow‐up adherence is critical for timely diagnosis and treatment, ultimately accelerating global efforts toward cervical cancer elimination.

## Abstract (highlights)

What's new?

Cervical cancer remains a significant burden despite being preventable, largely due to inadequate screening participation and poor follow‐up of high‐risk women. Here, the authors investigated follow‐up rates among women who tested positive for high‐risk human papillomavirus (hr‐HPV) infection in primary screening in Estonia. Data show that more than 57 percent of hr‐HPV‐positive women were lost to follow‐up screening within 12 months. Although most referred women completed colposcopy and treatment promptly, adherence was lower among older women and those in southern Estonia. The findings highlight key gaps in the screening pathway and inform efforts to improve follow‐up and cervical cancer prevention.

## Graphical abstract

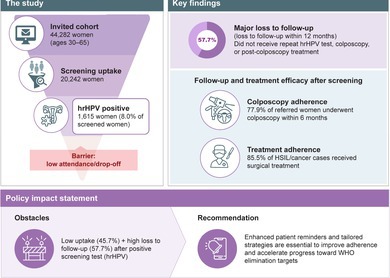

**Abbreviations**
ASCUS: atypical squamous cells of undetermined significance
C53: ICD‐10 code for cervical cancer
CCS: cervical cancer screening
CIN1: cervical intraepithelial neoplasia grade 1
CIN2+: cervical intraepithelial neoplasia grade 2 or higher
CIN3: cervical intraepithelial neoplasia grade 3
EHIF: Estonian Health Insurance Fund
HPV: human papillomavirus
hrHPV: high‐risk human papillomavirus
HSIL: high‐grade squamous intraepithelial lesion
ICD‐10: International Classification of Diseases, Tenth Revision
LBC: liquid‐based cytology
LSIL: low‐grade squamous intraepithelial lesion
N87: ICD‐10 code for cervical dysplasia
NAT: nucleic acid test
NILM: negative for intraepithelial lesion or malignancy
NOMESCO: Nordic Medico‐Statistical Committee
PP: percentage points
STROBE: strengthening the reporting of observational studies in epidemiology

## 1 Introduction

Cervical cancer was the first cancer to be targeted for elimination worldwide. The World Health Organization (WHO) global strategy sets an ambitious goal of eliminating cervical cancer through HPV vaccination and screening. Although cervical cancer is preventable, a significant number of new cases and deaths still occur in Europe [1, 2].

For most women, screening remains the primary mode of cervical cancer prevention. Challenges related to screening may include inadequate coverage, suboptimal uptake, and instances of missed, delayed, or insufficient follow‐up for abnormal primary screening test results. By 2023, 45 out of 47 European countries had implemented cervical cancer screening, with 23 countries providing well‐established population‐based screening [3]. Uptake of screening varies [4] and barriers have been comprehensively described [5]. There is an extensive body of research exploring ways to improve participation in screening programs [6].

Implementing a screening program is not merely offering a primary screening test, but rather a complex process that extends throughout a woman's life [7]. The screening pathway relies on the diagnosis and treatment of precancerous lesions. Globally, few studies have been published on the participation of organized HPV‐based cervical cancer screening pathways [8, 9, 10]. A recent review reported loss to follow‐up rates ranging from 4% to 75% among women with abnormal cervical cytology results [11], and approximately 12% of invasive cervical cancers can be attributed to a poor follow‐up of abnormal results [12].

Estonia's historically low cervical cancer screening uptake (less than 50%) and reliance on inefficient cytology‐based primary screening until 2020 [13], is associated with the high age‐standardized (new European standard population) incidence rate of 16.8 and mortality rate of 7.3 per 100,000, compared to the EU‐27 average incidence rate of 11.7 and average mortality rate of 5.3 per 100,000 in 2022 [14]. To date, no data have been published on the adherence to the Estonian hrHPV‐based screening pathway.

We aimed to map Estonia's HPV‐based cervical cancer screening pathway from primary screening test uptake, diagnosis and treatment of pre‐cancer and follow‐up at 12 months from the initial screening using electronic healthcare records for women invited to screening from January 1, 2021, to December 31, 2022.

## 2 Methods

### 2.1 Setting

**FIGURE 1:**
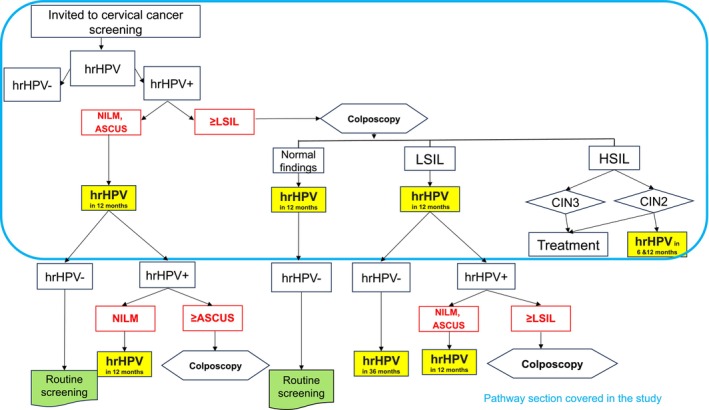
Cervical cancer screening pathway in Estonia (2021–2024). ASCUS, atypical squamous cells of undetermined significance; CIN 2, cervical intraepithelial neoplasia grade 2; CIN3, cervical intraepithelial neoplasia grade 3; HSIL, high‐grade squamous intraepithelial lesion; LSIL, low‐grade squamous intraepithelial lesion; NILM, negative for intraepithelial lesion or malignancy.

Estonia has had an organized cervical cancer screening program since 2006, initially based on Pap smears, offered every 5 years to 30–55‐year‐old women, who were covered with national health insurance. In 2021, the program transitioned to use of a high‐risk HPV nucleic acid amplification test (hrHPV NAAT) as the primary screening method. It also expanded coverage to non‐insured women and to the 30–65 age group, maintaining a 5‐year screening interval [15, 16]. Invitations are sent personally by email as well as to the digital patient portal. SMS reminders are sent to women who have not participated, 6 months after the beginning of the calendar year. Starting in 2024, opt‐in self‐sampling is part of a nationally organized screening program (women can order a kit through a web page). The cervical cancer screening pathway in Estonia is shown in Figure 1.

### 2.2 National Screening Algorithm

The cervical cancer screening pathway in Estonia involves triaging using results from primary hrHPV DNA testing and reflex liquid‐based cytology (LBC) for HPV‐positive cases. Women who are hrHPV‐positive but have cytology test results as negative for intraepithelial lesion or malignancy (NILM) or atypical squamous cells of undetermined significance (ASCUS) are advised to attend a follow‐up hrHPV test within 12 months. Women who are hrHPV‐positive and have an LBC result of low‐grade intraepithelial lesion (LSIL) or worse are referred for colposcopy by the healthcare provider who receives screening test results. According to the national recommendations on management of cervical intraepithelial neoplasia, hrHPV‐negative women return to routine screening. Individuals with LSIL confirmed on biopsy or colposcopy diagnosis of LSIL were recalled within 12 months for a follow‐up hrHPV test. If the hrHPV test 12 months after the diagnosis of LSIL is negative the next triage will be in 36 months. Women with colposcopy and biopsy‐confirmed findings of high‐grade cervical intraepithelial lesion (HSIL) receive appropriate treatment and are invited for follow‐up hrHPV testing at 6 and 24 months. Cases with HSIL (CIN2) may be monitored rather than immediately treated, particularly in women planning pregnancy in the near future, according to national recommendations on the management of cervical intraepithelial neoplasia. In this case the individuals will be referred for the follow‐up hrHPV test in 6 months and 12 months, and if both tests are negative the next triage will be in 36 months. It is recommended that women with diagnosed HSIL (CIN3) undergo treatment. There is no centralized monitoring or reminder system for follow‐up care. Women are responsible for scheduling appointments for follow‐up care.

### 2.3 Study Design and Data Sources

This retrospective cohort study utilized data from Estonia's nationwide and population‐based electronic health care system between January 1, 2021, and March 31, 2024, focusing on women who were invited based on year of birth to screening from January 1, 2021, to December 31, 2022.

#### 2.3.1 Estonian Health Insurance Fund

We analyzed data from the Estonian Health Insurance Fund (EHIF), the entity responsible for Estonia's nationwide, population‐based, and universal tax‐funded healthcare insurance.

The EHIF established in 2001 maintains a nationwide complete record of the health care services provided and reimbursed. Diagnoses were defined according to the International Classification of Diseases, tenth revision (ICD‐10). The EHIF database records sex, age, health care utilization information (dates of service, codes of services [procedures, tests, treatments, diagnoses codes]), and date of death.

#### 2.3.2 Analytical Cohort

The analytical cohort was established as part of a broader initiative to advance epidemiologic research using routinely collected electronic health and billing data in Estonia. The EHIF maintains comprehensive records of reimbursed health services, covering approximately 95% of the national population [17], which served as the sampling frame. Individuals insured by EHIF are broadly representative of the general population in terms of demographic and regional characteristics. From this frame, a simple random sample comprising 30% of insured individuals was drawn using EHIF's internal random sampling algorithm, ensuring that each person had an equal probability of inclusion. A robust and representative foundation for diverse health research projects is thereby provided by this cohort.

#### 2.3.3 Study Cohort

From this analytical cohort, we identified women aged 30–65 years who were invited to the cervical cancer screening program in 2021 (women born in 1956, 1961, 1966, 1971, 1976, 1981, 1986, and 1991) and 2022 (born in 1957, 1962, 1967, 1972, 1977, 1982, 1987, and 1992) formed our study cohort [18]. Data on women who were diagnosed with cervical cancer (ICD‐10 codes: C51, C52, C53, C54, C55) or carcinoma in situ (ICD‐10 D06) before January 1, 2021, were excluded. For each woman, all health claims from the EHIF were obtained from January 2021 to March 31, 2024.

The Population Register is a unified database of Estonian citizens and foreign nationals with residence permits. Population Registry data were used to identify study participants' education, ethnicity, marital status, and region of residence [19].

Data were linked through unique personal identification codes issued to every citizen in Estonia.

### 2.4 Study Outcomes

The primary outcome was the proportion of women with a positive hrHPV screening test who were lost to the first step follow‐up within 12 months. This is defined as the number of women who, within 12 months of their initial positive screen, did not attend a 12‐month repeat hrHPV test or undergo colposcopy. The extended primary outcome was the proportion of women with positive hrHPV screening tests who were lost to follow‐up within 12 months and was defined by two conditions: (1) not attending a 12‐month repeat hrHPV test or undergoing colposcopy, or (2) undergoing colposcopy but not receiving cervical disease treatment or a 12‐month repeat hrHPV test.

The secondary outcomes included (i) screening uptake (the proportion of women eligible for cervical cancer screening screened in the specified years), defined as those who were invited and participated in the cervical cancer screening; (ii) hrHPV positivity, defined as those who had hrHPV test and LBC service codes on the same health care bill; (iii) colposcopy uptake: the proportion of women who underwent colposcopy within 6 months among those with both hrHPV positivity and cytology results representing LSIL or more severe abnormalities in accordance with national screening guidelines, identified as women with hrHPV and LBC service codes on the same health care bill followed by a colposcopy code on a subsequent bill within 6 months.

The tertiary outcomes were (iv) the loss to follow‐up post‐colposcopy defined as the proportion of women who did not receive cervical disease treatment or a 12‐month repeat hrHPV test; (v) the proportion of women diagnosed with HSIL or cervical cancer following colposcopy, defined as women diagnosed with cervical intraepithelial neoplasia (ICD‐10: N87.1, N87.2 HSIL), carcinoma in situ (D06), or cervical cancer (C53 cervical cancer); and (vi) the proportion of women with HSIL or cervical cancer treated within 3 months after colposcopy, defined as women having treatment codes on the bill, following colposcopy.

There are limited data to guide clinicians for colposcopy timing and they are mostly based on expert consensus [20]. Several national organizations recommend target timeframes for colposcopy, varying from 2 weeks to 12 months after an initial screening test based on the severity of the abnormal results (LBC) [21, 22, 23]. The 6‐month time frame for colposcopy was chosen as it is advised not to exceed a 180‐day period to perform colposcopy for LSIL [1]. Prompt treatment of HSIL is generally recommended to prevent progression to invasive cervical cancer [24, 25]. Most guidelines do not specify the timeframe within which the treatment must occur [26]. We selected a 3‐month follow‐up interval based on clinical best practices and existing literature emphasizing the importance of early intervention to effectively manage and mitigate the risk of invasive disease [27].

While screening uptake was analyzed descriptively to contextualize participation, the primary analytical focus of this study was the follow‐up adherence among hrHPV‐positive women.

### 2.5 Statistical Analysis

For the assessment of the primary outcome, we assessed all diagnostic and therapeutic procedures related to cervical intraepithelial neoplasia (LBC, hrHPV test, colposcopy, loop electrosurgical excision, biopsy, hysterectomy, cervical conization, cervical ablation, cervical amputation and extirpation, hysterectomy) occurring within 12 months after the index HPV‐positive result. We allowed these procedures to occur from 9 months up to 15 months from testing (i.e., grace period).

We classified cohort members taking up screening (defined by having health care claim with the cervical cancer screening hrHPV test service code), testing hrHPV positive (having hrHPV test and LBC service codes on the same health care bill), having colposcopy (defined by EHIF service codes), and having cervical pre‐cancer treatment (defined by EHIF service codes). The data from 2 years (2021, 2022) were merged for this analysis.

The number and proportion of women were calculated for each step of the pathway. Proportions are reported for all steps of the pathway so that the number of women reaching a specific stage served as the denominator at the subsequent stage.

Descriptive analyses of age group, education, marital status, and nationality were performed for the study cohort and presented as proportions of the total. Follow‐up continued until the predefined study end date (31 March 2024) or ended earlier if an outcome of interest, cancer diagnosis, or death occurred first.

Categorical variables were presented as frequencies and percentages. Participation rate was defined as the proportion of eligible individuals within each category who attended the cervical cancer screening (test), calculated as the number of attenders divided by the total number of individuals in that category. A reference category was specified for each variable, and all other categories were compared against this reference. Rate differences were calculated by subtracting the rate in the reference category from the rate in each comparison category and were presented as absolute percentage‐point differences with 95% confidence intervals (CIs). Chi‐square test was used to assess if the observed differences in participation rates were statistically significant, and two‐sided *p* values were reported.

We conducted univariate logistic regression analyses to assess the association between participant characteristics and loss to follow‐up. In these analyses, loss to follow‐up was the dependent variable (coded as 1), with attendance coded as 0. All participant characteristics were initially included to explore potential associations and to estimate odds ratios (ORs) along with their 95% CIs.

For the multivariable logistic regression model, variables that showed a statistically significant association (*p* < 0.05) in the univariate analyses were retained. From this multivariable model, we then estimated adjusted odds ratios (AORs) and their 95% CIs.

Statistical analyses were performed using Rstudio software Version 2024.12.1 + 563 (2024.12.1 + 563), Copyright (C) 2025 by Posit Software, PBC.

This study follows the Strengthening the Reporting of Observational Studies in Epidemiology (STROBE) reporting guideline [28].

## 3 Results

### 3.1 Participants

The study sample was a random sample (*n* = 44,282) of women invited to undergo screening in 2021 and 2022. The analytical cohort comprises 20,242 women who participated in the cervical cancer screening in their respective years (2021: *n* = 9492; and 2022: *n* = 10,750) (Figure S1).

Women participating in the screening were largely representative of the target population for cervical cancer screening in Estonia. The largest age group was 29–39 (33.2%), followed by 40–49 (27.5%) and 50–59 (23.8%). Most participants had secondary (46.9%) or higher education (45.5%). Approximately half of the participants were married (47.2%). Participation rates increased from 2021 (42.7%, 95% CI: 42.0%–43.4%) to 2022 (48.7%, 95% CI: 48.0%–49.4%). Participation differed across sociodemographic groups (Table 1).

**TABLE 1:** Participation in cervical cancer screening by sociodemographic characteristics, Estonia 2021–2022.

Variable	Women participated in the screening, *N* = 20,242 (*n*)	Women not participated in screening, *N* = 24,040 (*n*)	Total women in group	Participation rate %	Absolute rate difference vs. ref (95% CI)	*p*
Age groups						
29–39	6723	7342	14,065	47.8	−2.2 (−2.9 to 1.5)	< 0.001
40–49	5568	5558	11,126	50.0	Ref	
50–59	4810	5916	10,726	44.8	−5.2 (−6.0 to −4.4)	< 0.001
60+	3141	5134	8275	38.0	−12.0 (−12.9 to −11.1)	< 0.001
Education						
Basic (≤ 9 years^a^)	1222	2208	3430	35.6	−15.8 (−17.6 to −14.0)	< 0.001
Secondary (12 years)	9490	12,222	21,712	43.7	−7.7 (−8.7 to −6.7)	< 0.001
Higher education	9217	8709	17,926	51.4	Ref	
Unknown	313	901	1214	25.8	−25.6 (−28.2 to −23.1)	< 0.001
Nationality						
Estonian	13,646	14,686	28,332	48.2	Ref	
Non‐Estonian	6483	8875	15,358	42.2	−6.0 (−6.9 to −5.0)	< 0.001
Unknown	113	479	592	19.1	−29.1 (−32.3 to −25.9)	< 0.001
Marital status						
Married	9561	10,107	19,668	48.6	Ref	
Single	5990	7301	13,291	45.1	−3.5 (−4.6 to −2.4)	< 0.001
Divorced	4252	5528	9780	43.5	−5.1 (−6.3 to −3.9)	< 0.001
Unknown	439	1104	1543	28.5	−20.2 (−22.5 to −17.8)	< 0.001

^a^ Of education.

Compared with women aged 40–49 years (reference), participation was significantly lower among those aged 29–39 (absolute difference −2.2 percentage points; 95% CI −2.9 to −1.5; *p* < 0.001). This decline continued in the older age groups. Women aged 50–59 had a lower participation rate than the reference group (absolute difference −5.2 pp; 95% CI −6.0 to −4.4; *p* < 0.001), and the lowest participation was observed among women aged 60 years and older (absolute difference −12.0 pp; 95% CI −12.9 to −11.1; *p* < 0.001).

Large educational differences were observed, with participation rates correlating positively with higher educational attainment. Compared with women with higher education (reference), participation was substantially lower among those with secondary education (absolute difference −7.7 pp; 95% CI −8.7 to −6.7; *p* < 0.001) and especially among those with basic education (absolute difference −15.8 pp; 95% CI −17.6 to −14.0; *p* < 0.001).

Regarding nationality, Estonian women served as the reference category and had the highest uptake. Participation was significantly lower among non‐Estonian women (absolute difference −6.0 pp; 95% CI −6.9 to −5.0; *p* < 0.001).

By marital status, married women had the highest participation (reference). Participation was significantly lower among single women (absolute difference −3.5 pp; 95% CI −4.6 to −2.4; *p* < 0.001) and divorced women (absolute difference −5.1 pp; 95% CI −6.3 to −3.9; *p* < 0.001).

### 3.2 Secondary Outcomes

During the study period, 45.7% (*n* = 20,242, 95% CI: 45.3%–46.2%) of invited women participated in primary cervical cancer screening (Table 1). Among these participants, 1615 (8.0%, 95% CI: 7.6%–8.3%) tested positive for hrHPV.

**FIGURE 2:**
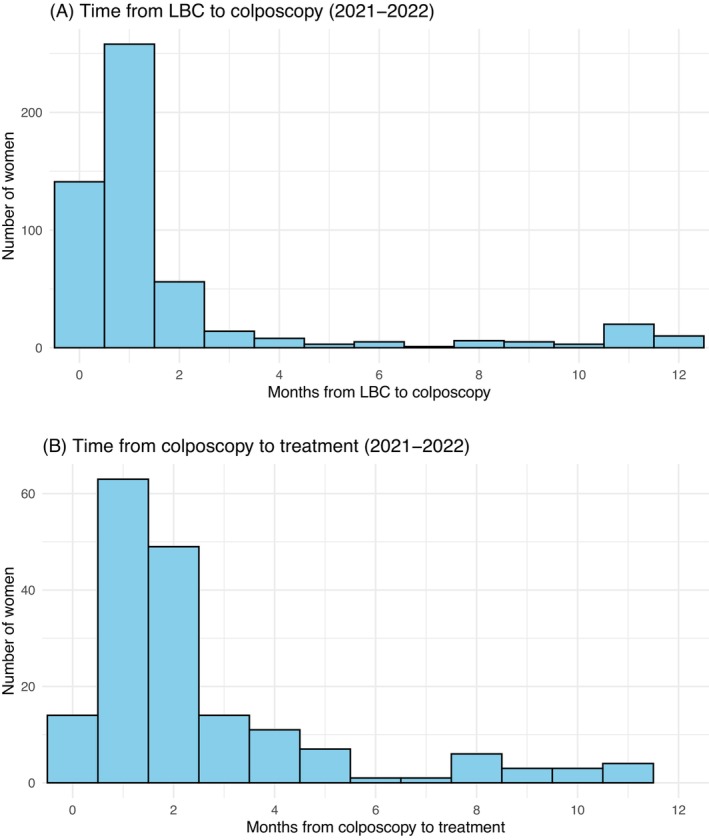
Time intervals in the cervical cancer screening pathway in Estonia (2021–2022) (Panel A: Time from positive primary screening test to colposcopy; Panel B: Time from colposcopy to treatment).

Of the 1615 hrHPV‐positive women, 530 (32.8%, 95% CI: 31.1%–35.7%) underwent colposcopy, consistent with the screening protocol where women with LSIL or more severe cytology are referred for colposcopy. Among these, 77.9% (*n* = 413, 95% CI: 74.2%–81.4%) underwent the procedure within 6 months of HPV testing. The frequency distribution of the time difference (in months) between a positive hrHPV test result and colposcopy is presented in Figure 2 (Panel A).

Two thirds (*n* = 355, 67.0%) of the women underwent colposcopy within the first 2 months of their positive screening test, with half (*n* = 263, 49.6%) of the colposcopies conducted within 1 month. There was a considerable spread in the distribution, with the time to colposcopy extending 6 months for some women.

Colposcopy examination identified LSIL/CIN1 in 13.2% (*n* = 69; 95% CI: 10.3%–16.2%), HSIL/CIN2 in 11.5% (*n* = 61; 95% CI: 8.9%–14.5%), and HSIL/CIN3 in 14.7% (*n* = 78; 95% CI: 11.8%–18.0%) of participants. Unspecified cervical intraepithelial neoplasia was identified in 12.6% (*n* = 65; 95% CI: 9.6%–15.3%), and invasive carcinoma in 0.1% (*n* = 6; 95% CI: 0.04%–0.25%).

Among women who underwent colposcopy within 2 months, 80.6% (*n* = 427; 95% CI: 76.9%–86.9%) had cervical biopsies. In 85.5% (*n* = 124; 95% CI: 78.7%–90.8%) of HSIL and cancer cases, surgical treatment followed colposcopy, with 74.2% (*n* = 92; 95% CI: 65.6%–81.6%) of these interventions occurring within 3 months. Figure 2 (Panel B) shows the frequency distribution of the time interval from colposcopy to treatment among the women receiving further intervention. More than half of the treatments (*n* = 106, 60.2%) occurred within the first 2 months, and most treatments (*n* = 136, 77.3%) occurred during the first 3 months after colposcopy. However, the distribution exhibits notable variability, with some women experiencing extended delays of up to nearly 1 year before treatment initiation.

### 3.3 Primary Outcomes

**FIGURE 3:**
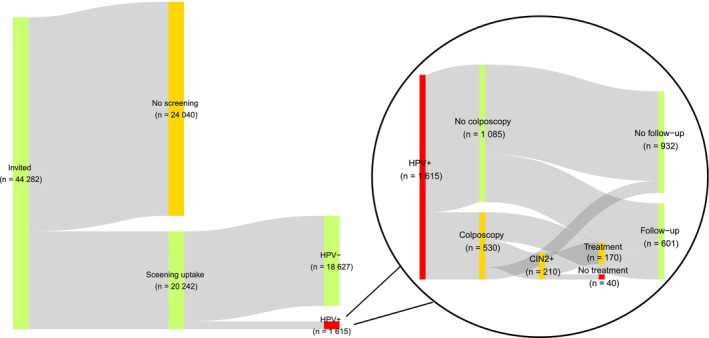
Pathway analysis of cervical cancer screening uptake and follow‐up among women screened in 2021–2022, Estonia. CIN2+, cervical intraepithelial neoplasia grade 2 or higher; HPV+, high‐risk human papillomavirus positive.

The proportion of women lost to initial follow‐up within 12 months, who did not attend a 12‐month repeat hrHPV test or undergo colposcopy, was 43.8% (*n* = 708). The extended primary outcome—the proportion of women with a positive hrHPV screening test who within 12 months either did not attend a repeat hrHPV test or undergo colposcopy, or who underwent colposcopy but did not receive cervical disease treatment or a repeat hrHPV test—was 57.7% (*n* = 932) (Figure 3).

### 3.4 Factors Associated With the Loss to Follow‐Up at Month 12 (Primary Outcome)

Region of residency and age were associated with 12‐month follow‐up non‐adherence among women with positive hrHPV test results (Table 2). Women residing in the South‐Estonian region were less likely to attend follow‐up compared to those in the Harju County (capital) region (adjusted OR = 1.77, 95% CI: 1.38–2.25). Additionally, a clear age gradient was observed, with older women consistently less likely to complete the 12‐month follow‐up. The AORs were 1.63 (95% CI: 1.26–2.10) for ages 40–49, 1.50 (95% CI: 1.13–1.98) for ages 50–59, and 2.0 (95% CI: 1.46–2.74) for ages 60 and above.

**TABLE 2:** Baseline characteristics among women testing hrHPV positive at cervical cancer screening in Estonia (2021–2022) and adjusted odds ratios for sociodemographic factors (age and region) linked to non‐participation in follow‐up at 12 months.

Variable^a^	HrHPV‐positive (*N* = 1615)		Lost to follow‐up (*N* = 708)		Retained in follow up (*N* = 907)			
	*n*	%	*n*	%	*n*	%	Adjusted OR^c^	95% CI
Age group (years)								
29–39	765	47.4	284	37.1	481	62.9	1	
40–49	365	22.6	179	49.0	186	51.0	1.63	1.26–2.10
50–59	277	17.2	131	47.3	146	52.7	1.50	1.23–1.98
≥ 60	208	12.9	114	54.8	94	45.2	2.00	1.61–2.74
Region								
Harju County	846	52.4	327	38.7	519	61.3	1	
Ida‐Viru County	175	10.8	85	48.6	90	51.4	1.39	1.00–1.93
West‐Estonia	185	11.5	78	42.7	107	52.8	1.15	0.83–1.59
South‐Estonia	392	24.3	208	53.1	184	46.9	1.77	1.38–2.25
Education								
Basic (≤ 9 years^b^)	138	8.5	60	43.5	78	56.5	0.96	
Secondary (12 years)	297	18.4	129	43.4	168	56.6	0.92	0.65–1.40
Vocational secondary (12 years)	428	26.5	197	46.0	231	54.0	1.01	0.70–1.22
Higher education	735	45.5	316	43.0	419	57.0	1	
Nationality								
Estonian	1189	73.6	526	44.2	663	55.8		
Non‐Estonian	417	25.8	176	42.2	241	57.8		
Marital status								
Married	495	30.7	224	45.3	271	54.7		
Single	649	40.2	270	41.6	379	58.4		
Divorced/widowed	438	27.1	198	45.2	240	54.8		

^a^ Missing data not shown. Highest proportion of missing data: marital status (2.0%), region (1.1%), education (1.0%), ethnicity (0.6%). No missing data for age group.

^b^ Years of education.

^c^ Adjusted for age group and region.

## 4 Discussion

### 4.1 Key Findings

#### 4.1.1 Follow‐Up Adherence Among hrHPV‐Positive Women

Our study reveals a significant challenge in the cervical cancer screening pathway in Estonia: 57.7% of women with a positive hrHPV result were lost to follow‐up. This substantial lack of adherence, particularly concerning given the critically low overall screening uptake of 45.7%, indicates a major discontinuity in the pathway that could lead to preventable precancerous conditions progressing into invasive cancer due to delayed or missed appropriate diagnostic and therapeutic interventions.

Older women, particularly those aged 60+, were substantially less likely to attend follow‐up. Women residing in the South‐Estonian region were less likely to complete follow‐up compared with those in Harju County (Table 2).

While recent reforms in Estonia's cervical cancer screening program, implemented in 2021, Rigby et al. [29] have demonstrably improved screening coverage—evidenced by an increase from historically low uptake (less than 50%) to 65% in 2024 through expanded eligibility, diversified test provision (including family physicians and self‐sampling), and the shift to primary HPV testing—achieving the full preventive potential of these efforts necessitates ensuring that women complete the entire screening pathway.

#### 4.1.2 Progression Along the Cervical Cancer Screening Pathway

Among the screened women, the positivity rate for hrHPV (8.0%) was within the expected range for HPV‐based screening programs [30, 31], and was consistent with a recently published population‐based study from Estonia conducted in 2021, which reported a high‐risk HPV prevalence of 8.8% [32].

In Estonia, 33% of HPV‐positive women were referred for colposcopy, which is comparable to international data: 35% in Australia [33], 39% in the Netherlands [9], and 35%–49% in Sweden depending on the screening protocol [9]. However, differences in triage algorithms and healthcare systems may limit direct comparability, and not all women needing colposcopy may have reached it.

In the current study, reflex cytology diagnosed LSIL+ in 32.8% of hrHPV‐positive women, which is comparable to the 39.1% reported in a Dutch cohort [9]. Following colposcopy, HSIL was identified in 9.0% of patients in the hrHPV‐positive cohort. This observed HSIL rate of 9.0% is lower than the 15.1% documented in the ESTAMPA study (Latin America) but closely aligns with findings from a recent US study, which reported a rate of 7.6% [34, 35].

Among women attending colposcopy, 82.3% underwent biopsy [36]. The notably higher biopsy rate in Estonia than in England (49.1%) is unclear but may involve differences in clinical practice patterns or diagnostic thresholds.

#### 4.1.3 Socio‐Demographic Disparities

Our findings reveal significant and systemic disparities in cervical cancer screening uptake across all examined sociodemographic factors in Estonia. The 40–49 age group exhibited the most effective screening uptake, exceeding the participation rates of both younger (29–39 years) and older cohorts, suggesting successful engagement by current screening initiatives within this demographic. Conversely, the most critical shortfall was observed among older women, as the rate among the 60+ age group was substantially lower, reflecting a persistent under‐representation of this cohort in screening.

Large differences were also observed across education levels. Education is often utilized as a reliable proxy for socio‐economic status because educational attainment is strongly and consistently correlated with long‐term factors like income, occupational prestige, and health literacy, which collectively determine access to and utilization of health services. Consistent with this, women with basic education participated at a rate substantially lower than those with higher education, indicating significant health literacy barriers. Furthermore, Estonian women participated notably more frequently than non‐Estonian women, suggesting structural barriers related to language and cultural access. These participation gaps represent substantial missed screening opportunities nationally and align with previous research indicating widening disparities between Estonian and non‐Estonian women despite overall improvements in screening uptake [37]. Although our data did not include direct measures of socioeconomic status, regional variation may partly reflect underlying socioeconomic and infrastructural differences. Women residing in the South of Estonia region and older women were underrepresented in the follow‐up [37, 38].

These findings underscore the persistent influence of structural and individual level barriers on the care continuum, even in settings with universal health coverage.

Participants residing in rural areas were disproportionately affected by missed follow‐up. Previous studies have shown that limited access to specialist services, greater travel distances, and underdeveloped public transportation infrastructure are well‐documented contributors to reduced engagement with follow‐up care in both low‐ and high‐resource settings [39, 40, 41]. Although services may be nominally free, indirect costs—such as travel expenses, time away from work, and caregiving responsibilities—can serve as significant disincentives to timely care‐seeking [42].

Geographic disparities often intersect with socioeconomic inequalities. Lower household income is associated with reduced health‐seeking behavior, particularly when additional costs—however modest—are perceived as burdensome [43]. Studies from various countries have demonstrated that offering extended clinic hours, mobile clinics, and a single visit approach (“see‐and‐treat”) enhance follow‐up adherence among disadvantaged populations [44, 45, 46, 47]. A Canadian study found that older women were significantly less likely to return for follow‐up care after an initial abnormal test result, a pattern that has been reported in other population‐based studies across diverse screening settings [48]. Reduced follow‐up among older women is influenced not only by individual factors such as low perceived risk, digital literacy, and comorbidities, but also by provider communication and system coordination. How information is delivered and understood strongly impacts patient engagement and follow‐up adherence [49].

These challenges are particularly relevant in the context of Eastern Europe, where cervical cancer incidence and mortality remain among the highest in the WHO European region—age‐standardized incidence exceeding 20 per 100,000 and mortality rates of 5 per 100,000 in several countries [50]. In Estonia, invitations for screening are primarily delivered electronically—via email, the national digital patient portal, and SMS reminders for non‐attenders within the first 6 months. However, dependence on digital communication may limit follow‐up participation among older women and those with lower digital literacy. Moreover, rurality may further compound access barriers, as colposcopy services are largely concentrated in urban centers.

Older women may be especially vulnerable to unclear or overly technical explanations about HPV, particularly when care is fragmented or providers lack confidence. Inconsistent messaging can undermine trust and reduce follow‐up attendance. Additionally, past stigmas regarding sexual health may make older women less likely to seek clarification unless they foster a respectful and supportive environment [51]. However, many clinicians receive little formal training in communication skills, particularly in addressing sensitive topics like HPV in older or postmenopausal women [51, 52].

To improve uptake, attention should be focused on groups that remain underserved by the screening program. Moving beyond a “one‐size‐fits‐all” strategy towards a more tailored approach could enhance overall participation in the cancer continuum of care.

At the moment, the formal reminder system for screening uptake currently relies on passive, automated channels, including electronic invitations and automated SMS notifications. Direct, person‐to‐person outreach, such as phone reminders, is not routinely implemented. However, broader awareness and promotion efforts are utilized: pharmacy staff routinely remind eligible women about the screening opportunity during their visits, and periodic national media campaigns are conducted using multiple communication channels, including television, social media, posters, and radio, to promote screening participation and increase public awareness.

#### 4.1.4 Global Implications of Follow‐Up Challenges

The findings from this study within the Estonian cervical cancer screening program illuminate a pervasive challenge encountered internationally. The substantial proportion of women lost to follow‐up underscores a critical vulnerability in the continuum of care, demanding global attention and the development of effective intervention strategies to ensure timely access to necessary diagnostic and therapeutic procedures.

The shortcomings identified in systematic reminder systems for women with positive hrHPV results, coupled with potential deficits in their understanding of the implications of such findings, resonate across diverse healthcare settings [53, 54]. The reliance on individual initiative for scheduling follow‐up appointments, particularly in the absence of clear and consistent communication regarding risk and the necessity of subsequent testing, likely contributes to suboptimal adherence worldwide. Addressing these informational and procedural gaps is paramount for optimizing screening program effectiveness on an international scale.

Comparative analysis revealed lower follow‐up rates in Estonia relative to regions such as Italy (79%), and similar to those reported in Australia (55%), and New Mexico (49%) and underscores the heterogeneity in screening program performance globally [55, 56]. This highlights the importance of context‐specific adaptations and the potential for learning from successful models that have been implemented elsewhere. Although the study did not directly examine follow‐up procedures, public awareness, or guideline adherence and understanding of recommendations, the findings highlight potential areas in which improvements could enhance the overall effectiveness of cervical cancer prevention and early detection programs.

Effective cervical cancer screening programs rely not only on accurate initial testing but also on robust systems to ensure appropriate follow‐up for women with positive results. Two critical challenges faced by the Estonian screening program that hinder optimal follow‐up are: (1) Lack of systematic mechanisms to remind women to return for necessary follow‐up appointments or repeat testing. This places a disproportionate burden on individuals to navigate a complex healthcare system, potentially leading to delays or loss to follow‐up; and (2) national guidelines lack standardized timeframes for key steps in the follow‐up process, such as the interval between abnormal test results and colposcopy, or the time to treatment for precancerous lesions. This ambiguity can result in variations in clinical practice and treatment delays, potentially compromising patient outcomes [53, 54].

### 4.2 Strengths and Limitations

This study provides a representative assessment of the real‐world implementation of the national screening pathway in Estonia, based on a random sample of women invited to cervical cancer screening in 2021 and 2022. A key strength lies in the use of comprehensive healthcare billing records, which inherently mitigate recall and social biases. The reliance on objective administrative data, rather than self‐reported information, enhances the accuracy and reliability of the findings. The integration of data from the EHIF and the Population Register enabled a detailed analysis of screening participation, testing modalities, treatment pathways, follow‐up adherence, and key patient characteristics.

Importantly, this study addresses a critical public health issue by highlighting weaknesses in follow‐up care for hrHPV‐positive women—a known bottleneck in achieving the full preventive potential of cervical cancer screening. As one of the few analyses focused specifically on follow‐up in the context of Estonia's screening program, the findings underscore the importance of strengthening system‐level interventions to improve adherence to post‐screening guidelines.

Notwithstanding these strengths, several methodological limitations should be considered. While the EHIF database offers extensive coverage of healthcare claims, the absence of certain granular variables, such as explicit HPV test results within the claims data, necessitates inferential assumptions. For instance, hrHPV positivity was inferred when both an HPV test and a LBC examination were documented within the same healthcare claim. However, given the standardized protocols for claims filing and reporting the care pathway, we consider the reliability of these inferences robust. Furthermore, the study lacked detailed information regarding specific LBC results, precluding the determination of the proportion of women requiring colposcopy (i.e., those with LSIL+ results). The potential for random data omissions within the EHIF and Population Register databases represents another limitation that could introduce a degree of imprecision in the reported findings. Finally, the relatively short follow‐up duration does not fully capture longer‐term outcomes, such as the cumulative incidence of subsequent follow‐up procedures or the eventual clinical trajectory of hrHPV‐positive women.

## 5 Conclusions

This nationwide study provides a comprehensive overview of the cervical cancer screening pathway in Estonia following the transition to hrHPV‐based primary screening. The findings highlight a moderate screening uptake and identify a significant challenge in the high proportion of women lost to follow‐up after a positive hrHPV test. This gap in the continuum of care warrants urgent attention to improve adherence to follow‐up recommendations and prevent the potential progression of precancerous lesions. A critical requirement for strengthening Estonia's cervical cancer prevention program is the establishment of a centralized national management system, overseen by a dedicated governing body. Such a structure would integrate the entire care pathway—from initial screening and reminders to colposcopy, pathology, and treatment services—thereby guaranteeing consistent coordination, standardized quality monitoring, and the timely implementation of necessary improvements.

## Author Contributions

**Aleksandra Šavrova:** conceptualization, writing – original draft, methodology, visualization, writing – review and editing. **Helen Jakoby:** visualization, formal analysis, software, validation, data curation, writing – review and editing. **Anna Tisler:** conceived the study idea, conceptualization, writing – original draft, review and editing. **Kaire Innos:** writing – review and editing. **Ülo Maiväli:** methodology, formal analysis, software, writing – review and editing, visualization. **Jana Jaal:** writing – review and editing. **Anneli Uusküla:** conceived the study idea, conceptualization, methodology, funding acquisition, writing – review and editing, writing – original draft, supervision.

## Funding

This work was supported by Estonian Research Council, grant PRG2218. The work of K.I. was supported by Estonian Research Council, grant PRG2543.

## Ethics Statement

The Research Ethics Committee of the University of Tartu approved the study protocol (Decision number: 385/M‐25, 18.12.2023).

## Conflicts of Interest

The authors declare no conflicts of interest.

## Supporting information


**Figure S1:** Flow diagram reporting exclusions and the final sample of 2021 and 2022 CC screening study cohort.

## Data Availability

The data that support the findings of this study are available from the corresponding author upon reasonable request.
