# Supplementary material for: Nationwide Study on the Cervical Cancer Screening Pathway in Estonia
Source: Int J Cancer. 2026 Apr 9;159(4):883–95. doi: 10.1002/ijc.70466 (PMC13284620; doi:10.1002/ijc.70466)

Title: **Nationwide study on the cervical cancer screening pathway in Estonia**

Aleksandra Šavrova , Helen Jakoby , Anna Tisler , Kaire Innos , Ülo Maiväli , Jana Jaal , Anneli Uusküla

Table of Contents

Supplementary Figure S1

Supplementary Figure S1. Flow diagram reporting exclusions and the final sample of 2021 and 2022 CC screening study cohort.

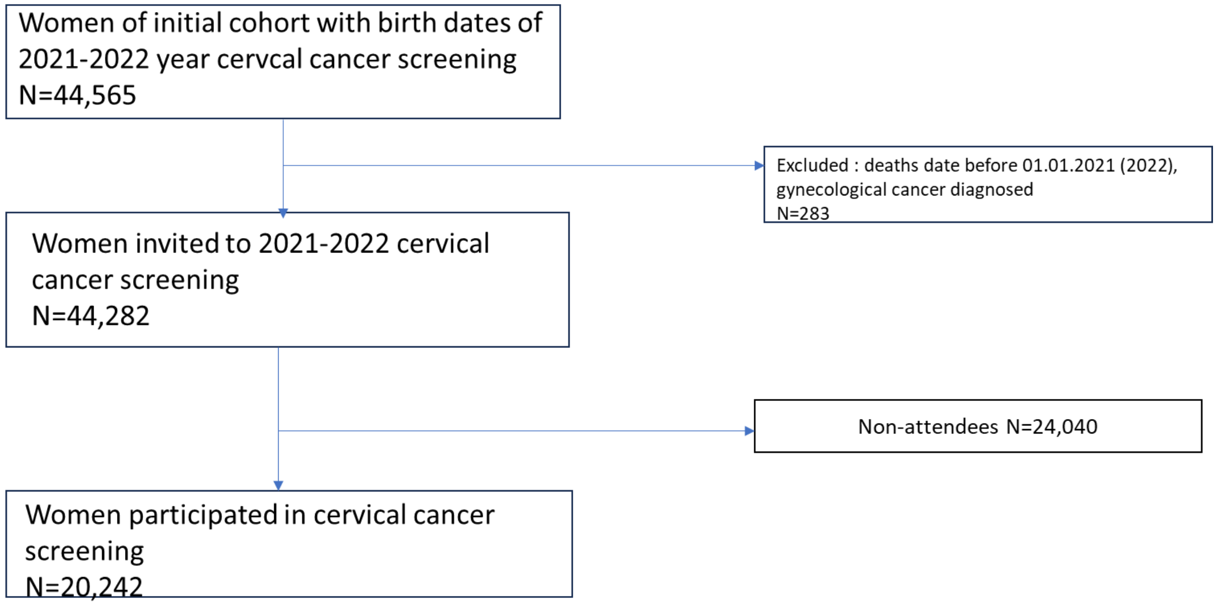

Supplement: Supplementary file 1 — Figure S1: Flow diagram reporting exclusions and the final sample of 2021 and 2022 CC screening study cohort. [file IJC-159-883-s001.pdf]
